# Aerial pruning mechanism, initial real environment test

**DOI:** 10.1186/s40638-017-0073-3

**Published:** 2017-11-10

**Authors:** Javier Molina, Shinichi Hirai

**Affiliations:** Department of Robotics, Ritsumeikan University BKC, 1-1-1 Noji-higashi, Kusatsu-shi, Shiga 525-8577 Japan

**Keywords:** Aerial robot, Skew-gripper, Grasping, Pruning

## Abstract

In this research, a pruning mechanism for aerial pruning tasks is tested in a real environment. Since the final goal of the aerial pruning robot will be to prune tree branches close to power lines, some experiments related to wireless communication and pruning performance were conducted. The experiments consisted of testing the communication between two XBee RF modules for monitoring purposes as well as testing the speed control of the circular saw used for pruning tree branches. Results show that both the monitoring and the pruning tasks were successfully done in a real environment.

## Background

In recent years, the popularity of multirotor helicopters has drastically increased to the point that almost everyone has heard the word drone. Low prices and a very competitive market have made possible the access to this technology either for fun or for research activities. Nowadays, the most popular application of multirotor helicopters is aerial video and photography; however, this activity is only for the purpose of capturing video and pictures from the environment without contact.

On the other hand, aerial manipulation focuses on the environment; basically, we can catalog this application in two main activities:

- Transportation
- Contact tasks

Load transportation deals with moving payload from one place to another using an aerial vehicle. It is commonly used a gripper to pick up the payload and move it to the target place; this task requires to control the stability of the aircraft which is affected by the payload [1–5].

Contact tasks using multirotor helicopters are used to interact with the physical environment to perform a specific task. In this case, the multirotor system is endowed either with a tool or with a manipulator. which allows it to execute the task. Examples of this research are: an Asctec Pelican quadrotor endowed with a custom-made manipulator for contact inspection [6], an aerial vehicle along with a couple of robotic arms for turning a valve using a human–machine interface [7] and a ducted-fan aerial vehicle for ultrasonic nondestructive structural inspection [8].

The examples mentioned above are clearly new applications related to multirotor helicopters. Considering that the stability of multirotor helicopters is affected by the payload during the process of grasping and moving and, the flying time is in most of the cases, a crucial factor to perform activities such as inspection by contact operations, we propose aerial manipulation only for the initial operation task. In other words, we propose to carry a tool to the point of interest, fix the multirotor using a gripper, perform the task and finally, return to the ground station. This allows the helicopter to reduce the energy consumption since it is only necessary to fly to the desired position and the rest of the operation will be performed by the tool without flying.

**Fig. 1:**
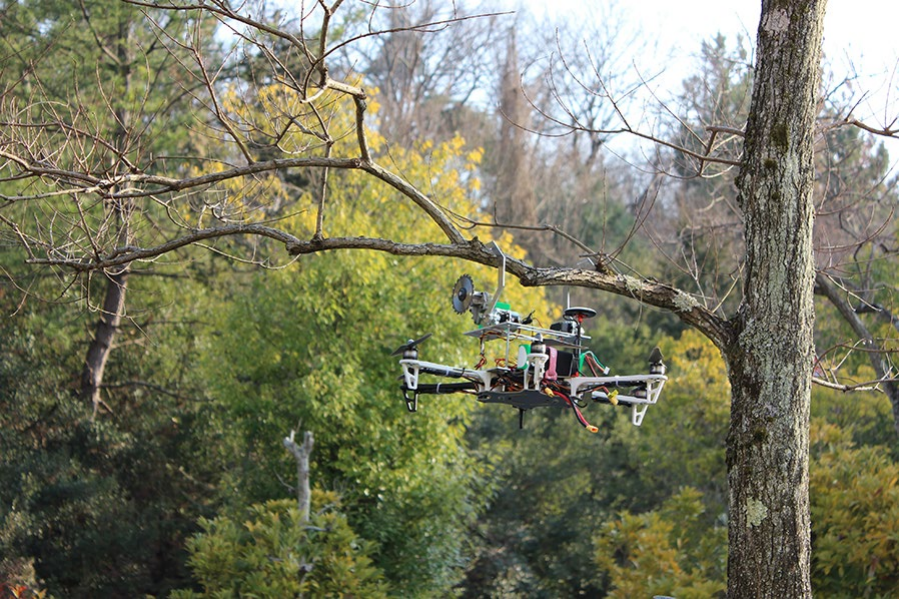
Scope. Aerial pruning robot

Particularly, we are interested in pruning tree branches using an aerial pruning robot as it is shown in Fig. 1; this is due to tree branches growing too close to power lines represent a potential hazard for the security of the residents as well as for the electricity supply. As an example, consider the case in which a tree branch hits one of the cables of a power line, it may cause an electrical arc producing sparks affecting the energy supply or even fire around the contact area [9]. In order to keep safety, tree branches must be kept away from electric power lines. Usually, to remove these tree branches, it is needed a person and a crane, the latter is to access the target and the former is to prune them with a specialized tool.

Pruning tree branches close to power lines represent a risk; this means that there is always the possibility of an accident caused by a high-voltage cable. Usually, the minimum required working distance for pruning trees close to a primary distribution line (between 750 and 150,000 V) and a transmission line must be 3 and 6 m, respectively. For a human worker, pruning these branches may become a difficult and hazardous task, that is, it is necessary to find a solution to perform such activity safety without direct human intervention in the task.

In this paper, we discuss two important tasks the aerial pruning robot should perform, communication with the user’s interface and pruning a real tree branch. First of all, a description of the aerial pruning robot is explained; next, we discuss the electronic interface and the PI control for the pruning process. We also give a brief introduction to the XBee modules and the interface with the microcontroller, and finally, we give some conclusions and future work.

## Aerial pruning robot workspace

**Fig. 2:**
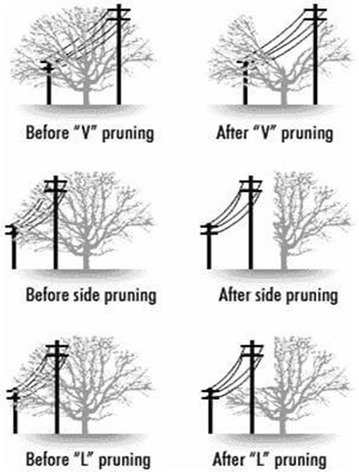
Tree trimming. Different ways to prune tree branches close to power lines, sketch taken from [15]

**Fig. 3:**
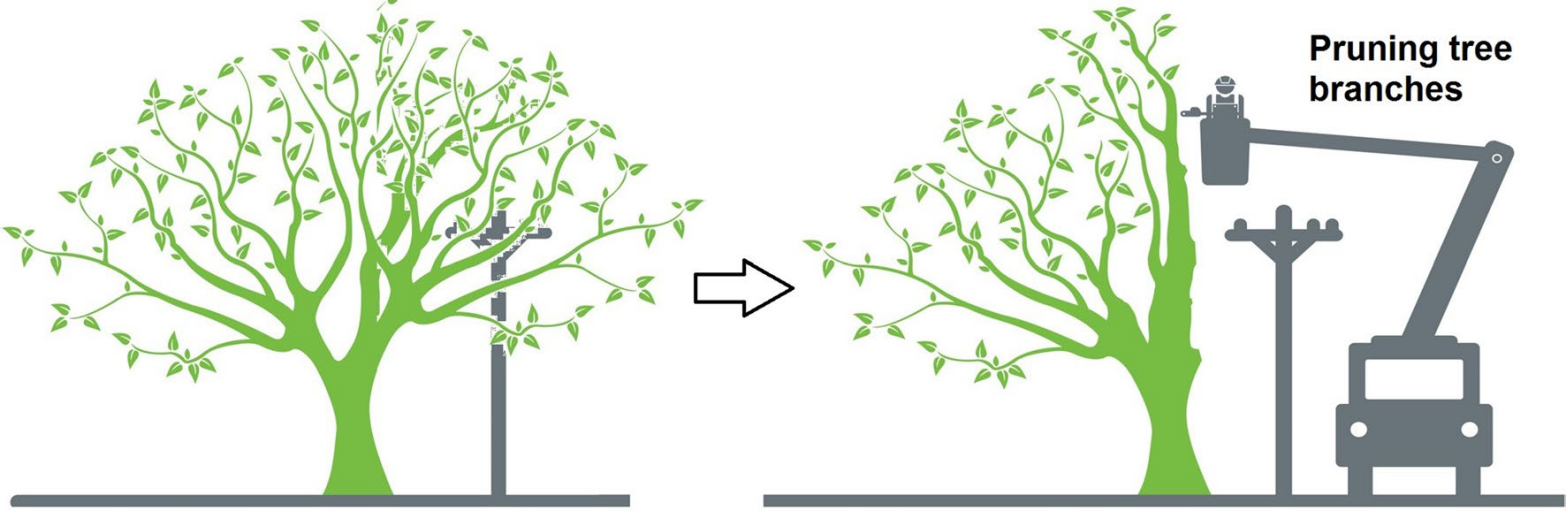
pruning_concept. A typical way to prune a tree branch close to an electrical power line. It is necessary a crane and a person to perform such activity, sketch taken from [16]

Generally speaking, there are three different ways to trim trees close to electric power lines [10]. These techniques are, “V” pruning, “L” pruning and side pruning, as they are shown in Fig. 2. In this project, we are focusing only in the side pruning technique; this is due to the mechanical characteristics of the multirotor and the grasping technique used to fix its body to the the target branch. Figure 3 shows a typical way to prune a tree branch close to power lines using the side pruning technique.

### Concept

**Fig. 4:**
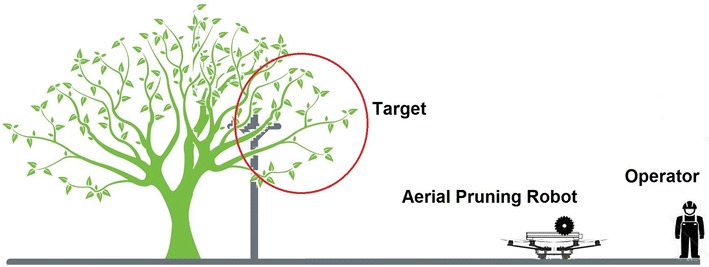
concepto_1. Aerial pruning robot, main concept

The idea we propose to prune tree branches close to power lines is to use a multirotor helicopter, a gripper and a circular saw to perform such activity. This new concept not only reduces the costs of using a truck with a crane but also reduces the human risks of a potential accident because of high voltage around the working area. Figure 4 shows the concept of the aerial pruning robot.

With this new idea, new challenges arise as they have to be solved in order to perform the complete task. In order to successfully complete the pruning task, the aerial pruning robot should accomplish four steps:

1. First of all, the aerial pruning robot should fly to the target branch.
2. When it is reasonable close to the target, a couple of claws-like grippers should close to grab the tree branch.
3. Once the tree branch was grabbed by the gripper and the complete body of the aerial pruning robot is hanging from the tree branch, the pruning mechanism, which is placed on the top of the multirotor, should start pruning the tree branch.
4. Finally, when the pruning task is done, the aerial pruning robot should come back to the home position.

**Fig. 5:**
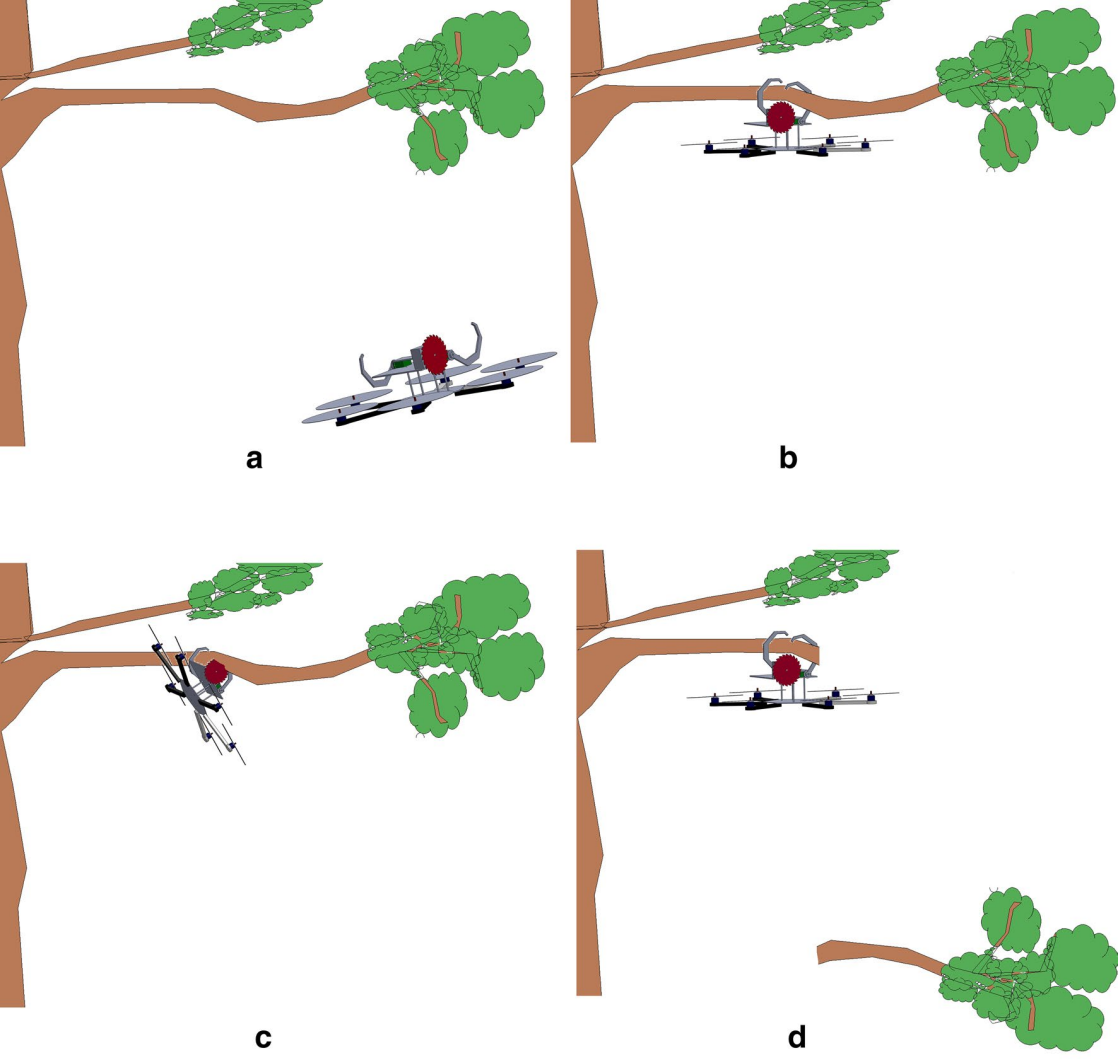
complete_process1. Sketch representative of the task to be performed. **a** The helicopter is flying to the target. **b** The helicopter is grasping the branch to be pruned. **c** Using a circular saw, the helicopter is pruning the branch taking advantage of the bracing technique. **d** The process of pruning is completed

Figure 5 shows the sketch representing the four steps the aerial pruning robot should complete.

## Mechanical description of the prototype

**Fig. 6:**
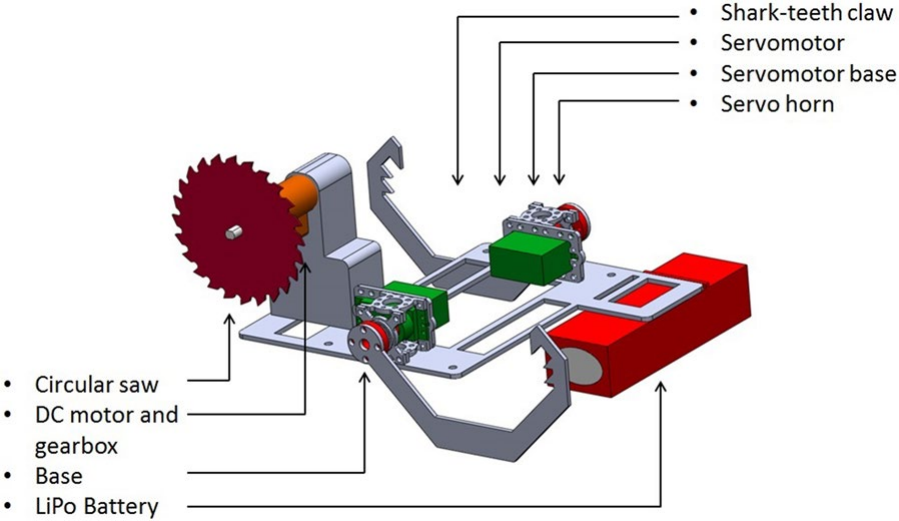
fig_description. Skew-gripper and pruning system

The robotic gripper that we are using in this research is composed by a couple of claws with teeth for grasping the tree branch firmly; since each claw is placed in different planes, we call this configuration “skew-gripper.” The pruning system is composed by a DC motor, a gear box, and a circular saw; Fig. 6 shows the complete CAD model of the mechanism, which is placed on the top of a multirotor helicopter.

**Fig. 7:**
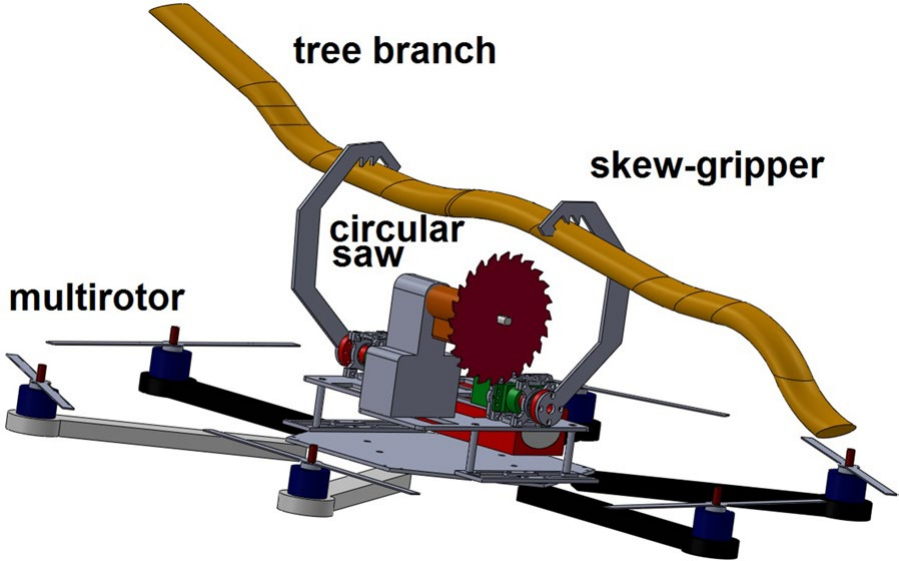
robot_branch. CAD model of the prototype hanging from a tree branch

By choosing a multirotor helicopter as a carrier of the pruning mechanism, specifically an hexarotor configuration, the complete mechanism is mounted on the top of it using a 3-mm aluminum base-plate. Figure 7 shows the CAD model of the prototype hanging from a tree branch using the skew-gripper as well as a circular saw ready to prune.

**Fig. 8:**
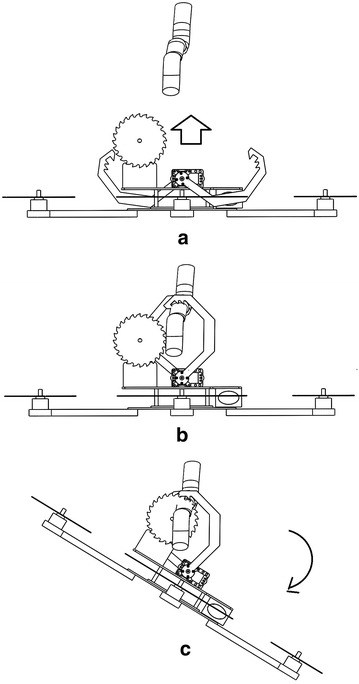
fliying_grasping_pruning. Transition between grasping and pruning process. In **a** the helicopter is flying to the tree branch. In **b** the tree branch is firmly grabbed and in **c** the helicopter’s body is rotating for pruning the tree branch

Figure 8 shows how the skew-gripper works. For grasping a tree branch, the couple of claws should open in an opposite direction, and when the tree branch is firmly grasped, there is no way for opening and closing anymore because of the shark-like teeth, which are well inserted in the tree branch. As the couple of shafts of the servo motors of the skew-gripper are aligned on the same rotational axis, the body of the helicopter with the circular saw is able to rotate along such axis creating a circular motion which is used to prune tree branches by means of a circular saw.

## Hardware description

### Characteristics of the circular saw

**Fig. 9:**
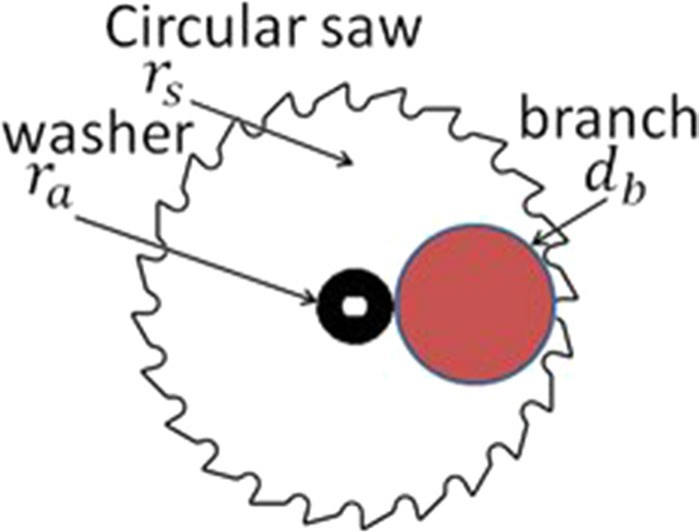
saw2. Relationship between the circular saw, the washer and the branch to be pruned

The skew-gripper can grasp a tree branch with a maximum diameter of 40 mm without any problem; however, the final goal is pruning; therefore, the diameter range, $d_{b}$ is set as:

1 $$\begin{aligned} a \le d_{b}\le b \end{aligned}$$

where *a* and *b* are the smallest and the largest diameters of the branch, respectively; this is due to the diameter of the circular saw introduce a restriction in the pruning task. In other words, the effectiveness diameter $d_{b}$ of the branch to be pruned using a circular saw is given by:

2 $$\begin{aligned} d_{b}=r_{s}-r_{a} \end{aligned}$$

where $r_{s}$ is the radius of the circular saw to be used and $r_{a}$ is the radius of the outer washer used to lock the saw to the actuator. Hence, the maximum diameter, *b*, of the branch to be pruned is determined by two factors, the circular saw and the outer washer. Figure 9 shows this relationship, and Table 1 shows the diameter of the circular saw to be used depending on the diameter of the branch the operator wants to prune.

On the other hand, the total weight of the aerial pruning robot is another factor which restricts the diameter $d_{b}$ of the branch to be pruned. That means, if the branch is too small, the total weight of the aerial pruning robot may bend it resulting in an inappropriate grasping or even the tree branch cannot support the weight of the helicopter and it may fall down. In order to prevent this possible issue, the minimum diameter, *a*, was determined experimentally in several branches resulting in $a=17$ mm, which can hold the helicopter safely.

**Table 1:** Relationship between the branch and the circular saw to be used

Diameter of the branch and circular saw	
Diameter of branch (mm)	Circular saw (mm)
17–29	85
30–39	110

### Controlling the rotational motion

**Fig. 10:**
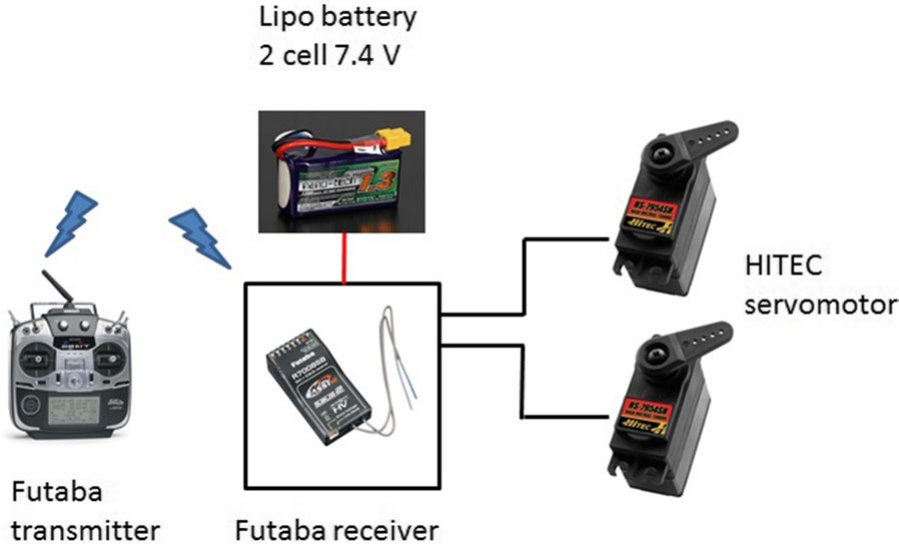
servo_system_control. Futaba transmitter and receiver for commanding the servo motors of the skew-gripper

In order to test the pruning system in a real environment, a hardware for controlling the swinging motion for pruning purposes was designed. A couple of high-torque servo motors from HITEC [11] and a Futaba 14SG radio transmitter and its respective receiver, the Futaba R7008SB [12], were chosen. These HITEC servo motors are used to move the skew-gripper for grasping as well as for creating the rotational motion for pruning tasks. In addition, for powering the servomotors and the receiver, a 7.4-V LiPo battery was used. Note that this battery is exclusively used for feeding the servomotors and the receiver; Fig. 10 shows these connections.

### Controlling the circular saw

**Fig. 11:**
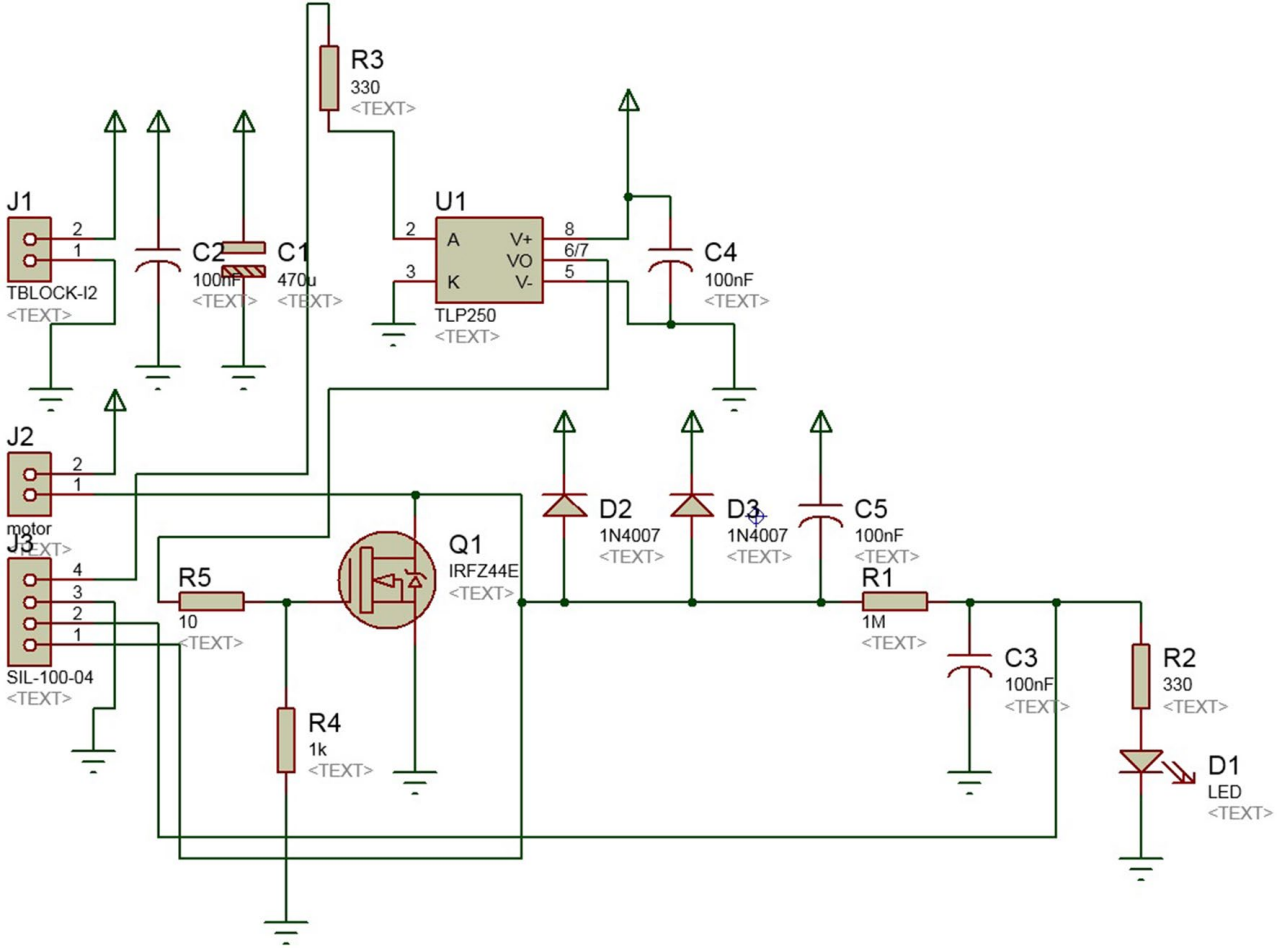
power_module. Power electronics module

The hardware designed for controlling the speed of the circular saw is composed by a power electronic module, which is used for commuting the circular saw’s DC motor, a back-electromotive force (back-EMF) module for sensing the motor’s speed, a PI controller programmed in an Arduino Uno board for regulating the speed of the circular saw and a RF XBee wireless module for transmitting the information of the pruning process to the operator. Figure 11 shows the schematic of the power system module, PIN 1 and PIN 2 of J1 connector are connected to ground and to 16.5V, respectively, PIN 1 and PIN 2 of J2 are connected to the terminals of the circular saw’s DC motor. From the connector J3, PIN 1 is the back-EMF signal, which is used for sensing the DC motor’s speed, PIN 2 was used as a back-EMF with a low-pass filter, but it was not worked well because of the noisy, and we decide to use the raw signal and design the low-pass filter separated as it will be explained later. Finally, PIN 4 is the PWM signal from the Arduino Uno board for the speed control the DC motor. The power electronic module is composed by a power MOSFET transistor together with a photo-transistor to protect it in case of over-current since the gate of this type of device is quite sensitive.

**Fig. 12:**
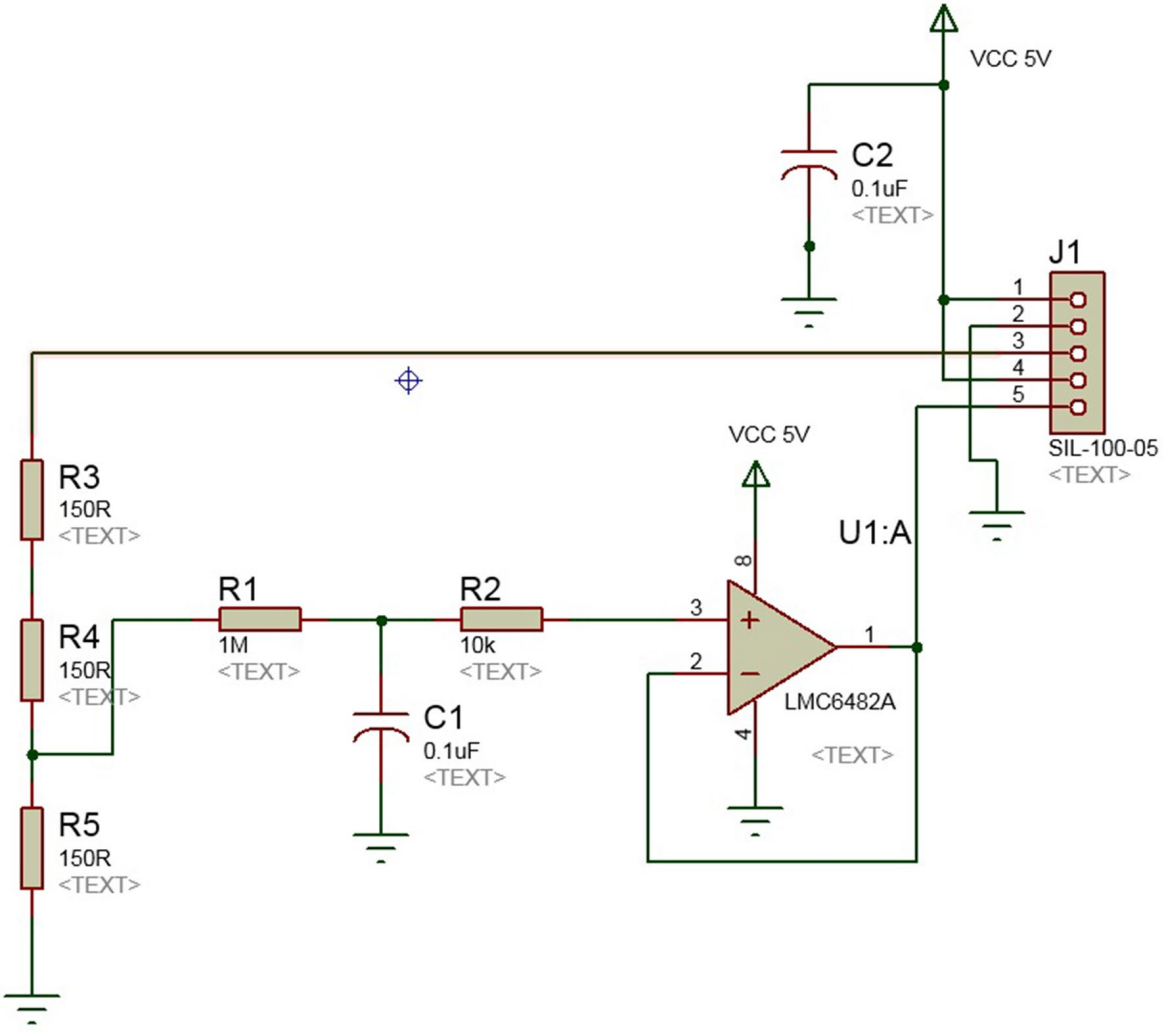
bemf. Module for sensing the back-EMF

Figure 12 shows the schematic of the back-EMF sensing module. This sensing module is for reading the back-EMF and is composed by a resistor divider to reduce the voltage from 16.5 to 5.5 V (J1 PIN 3), which comes from the drain pin of the MOSFET transistor; in addition, a low-pass filter is used to remove the noise from the DC motor. An operational amplifier configured as a voltage follower is used to reinforce the signal from the low-pass filter which also set the output voltage from 0 to 5 V (J1 PIN 5), suitable for an Arduino Uno board.

The wireless communication module, which is used to communicate the aerial pruning robot with the ground station, is composed by two XBee series 1 (S1) from DIGI International [13]. Some of the most important characteristics are summarized in Table 2.

**Table 2:** Specifications of the XBee S1 module

XBee S1 module	
Specification	Performance
Indoor/urban range	Up to 100 ft (30 m)
Outdoor RF line-of-sight range	Up to 300 ft (90 m)
RF data rate	250,000 bps
Serial interface data rate	1200 bps–250 kbps
Supply voltage	2.8–3.4 V
Transmit current (typical)	45 mA (@ 3.3 V)
ADC	6 10-bit ADC input pins
Operating frequency	2.4 GHz

**Fig. 13:**
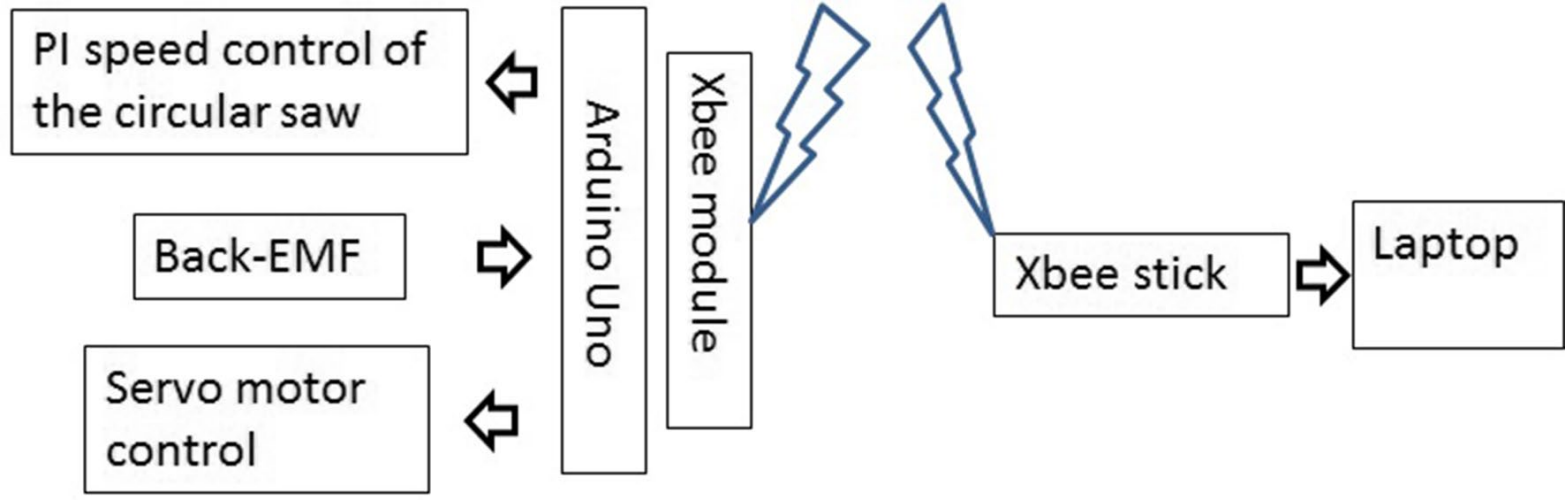
rcTransmitter. XBee modules for wireless communication

**Fig. 14:**
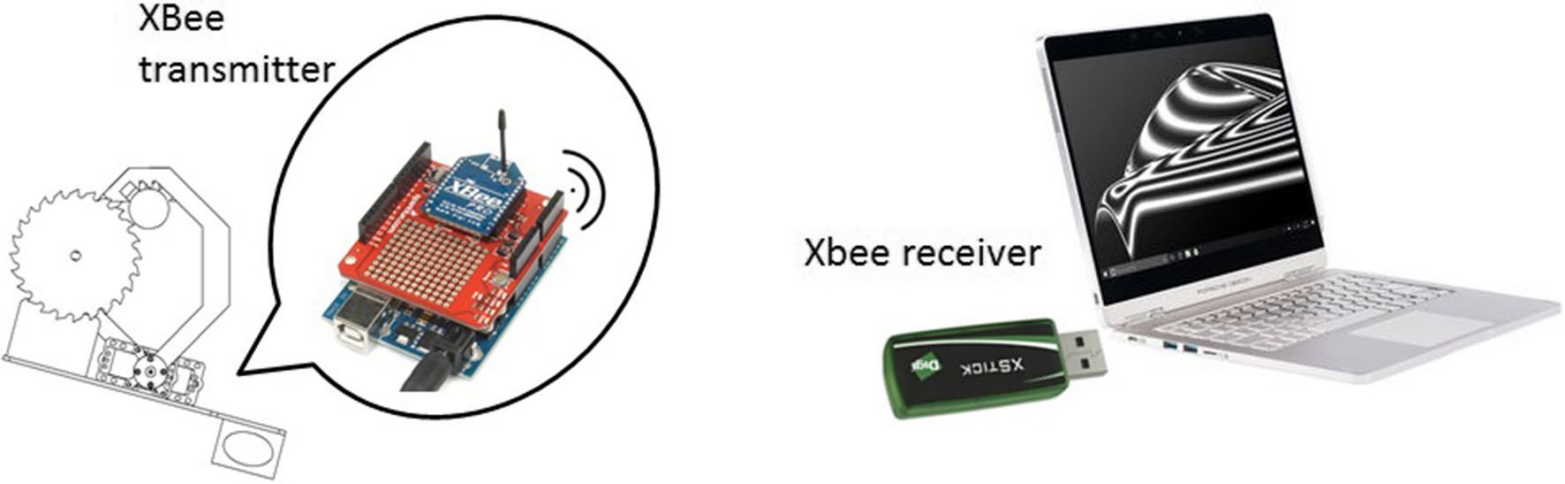
completop1. Modules for controlling the pruning process

One of the two XBee modules is connected to an XBee shield from Sparkfun [14], and this in turn is connected to an Arduino Uno board and hence sends the data to the computer placed in the ground station. The other XBee module, which plays the role of receiver, was connected to the USB port of the computer’s ground station for receiving the data from the aerial pruning robot. Unlike the XBee module used in the aerial pruning robot, the XBee module is connected to the computer’s ground station needless an interface to connect to it. This module is called “XStick,” and its shape is similar to an USB memory; however, it is used for wireless communication purposes. Figure 13 shows the wireless interface between the Arduino Uno and the computer for data transmission, and Fig. 14 shows the complete block diagram used for controlling and also for sending data to the computer in the ground station.

## Experimental results

In order to validate the performance of the pruning mechanism in a real environment, several experiments were performed. The aim of these experiments was to prove the effectiveness of the wireless communication between the aerial pruning robot and the ground station for monitoring the speed of the circular saw; in addition, the swinging motion for the pruning process produced by the couple of servomotors was also tested. For this experiment, a professional tipped-saw was selected as it will be described later.

### Pruning sequence

**Fig. 15:**
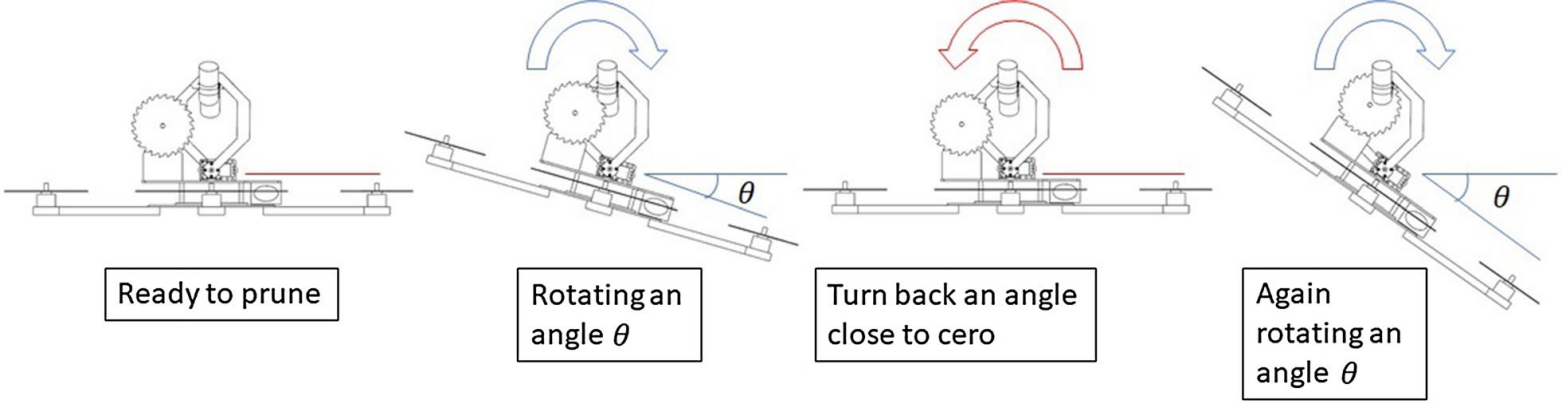
pruningSecuence2. Pruning sequence, from the left to the right, the pruning mechanism should rotate an angle $\theta$ and turn back to the initial position several times until the tree branch has been pruned

**Fig. 16:**
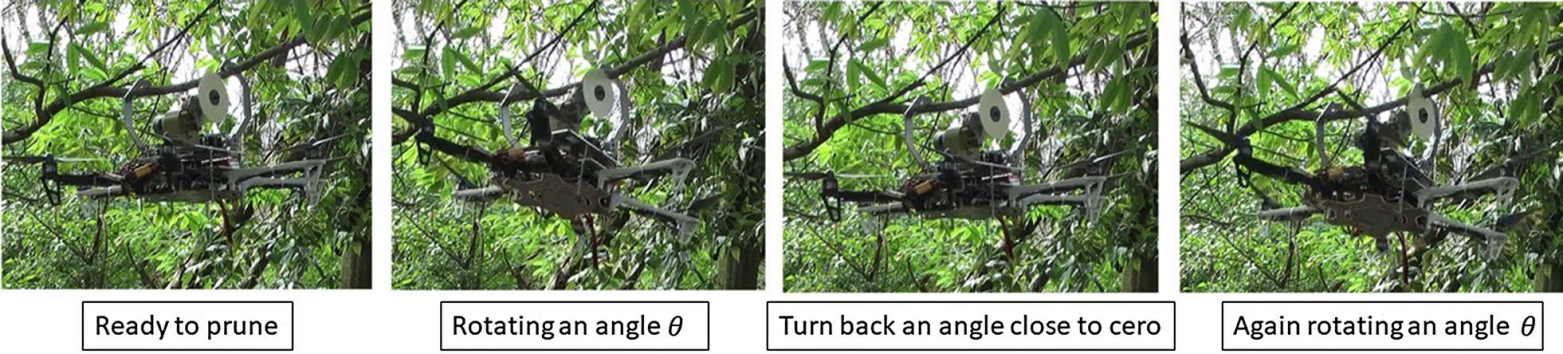
realProcess1. Real pruning sequence, from the left to the right, the pruning mechanism is idle and later, it starts pruning swinging repeatedly

In order to prune a tree branch ranging from 12mm to 40 mm, the pruning mechanism should start swinging to go through the tree branch progressively. In these initial tests, the operator decides when the pruning mechanism should turn back and goes again through the tree branch based on a visual inspection of the graphic of the circular saw’s speed provided by the computer placed in the ground station. For the sake of clarity, Fig. 15 shows the complete pruning task in four single steps, understanding that in a real situation, this swinging process should be repeated several times until the tree branch has been completely pruned. Figure 16 shows the sequence of pruning of the real prototype pruning a 17-mm-diameter tree branch. The circular saw used in these experiments has the characteristics mentioned as follows:

- Outer diameter: 100 mm
- Blade thickness: 1.3 mm
- Number of blades: 36
- Inside diameter (for attaching to the gear box): 20 mm

### Wireless communication and PI control performance

**Fig. 17:**
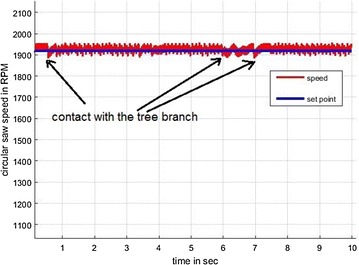
pruning2. PI speed control

Figure 17 shows the graphics of the performance of the pruning process; as it can be observed, the circular saw’s speed is constant with some fluctuation as a result of the contact force at the moment of pruning. The communication between the XBee modules was around 10 m without loosing the connection during the pruning task.

### Pruning results

#### Performance

**Fig. 18:**
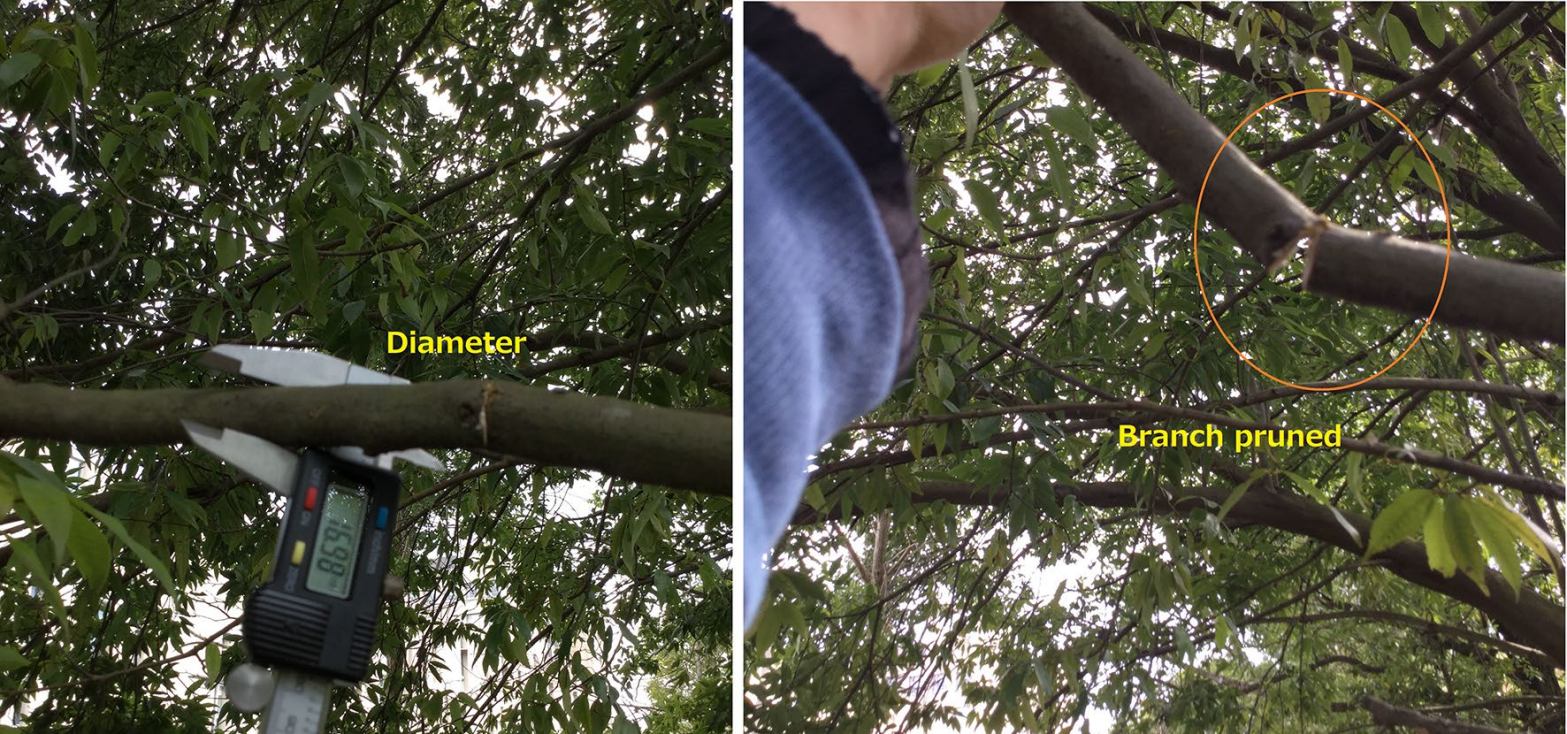
treeBranchPruned. Diameter of the tree branch used in these experiments, notice that the right side picture shows the cut produced by the circular saw

The tree branch used in this experiments as well as the performance of the pruning mechanism is mentioned in Table 3. From this table, one may appreciate that the pruning time is related to two important factors: the stiffness of the tree branch and the diameter. Figure 18 shows the tree branch pruned at approximately 90% of the total process.

**Table 3:** Main characteristics of the tree branch pruned in this experiment

Tree branch		
Length from the pruning area to the tip (m)	Diameter (mm)	Time for pruning
1.1	17	8 min

#### Energy consumption

Regarding the energy consumption of the whole system, the main source of energy consumption is the multirotor helicopter, and it consumes around 25 A during flying. On the other hand, the circular saw consumes only 4 A during the pruning process which takes around 8 min or less, depending of the diameter of the tree branch. In these experiments, a 5100-mAh 4S LiPo battery was used for powering the multirotor and the circular saw as well. This battery at full charge gives 16.8 V and should not go down less than 12.8 V at full discharge. For practical applications, we establish a boundary in 14.5 V to have enough time for landing in case the battery has achieved the minimal boundary and thus prevent a permanent damage. This range allows the operator to fly the multirotor around 7 min which is enough time for grasping and pruning at least, one tree branch.

## Conclusions

In this paper, the hardware description and some experimental results regarding pruning tree branches in a real environment using an aerial pruning robot were shown. Results obtained show that the PI control implemented to control speed of the circular saw was helpful for pruning a real tree branch. In addition, the wireless communication between the aerial pruning robot and a ground station has shown that it is possible to follow the pruning task monitoring the performance of the speed of the circular saw to avoid possible accidents. In the future, there are some necessary improvements to increase the performance of the pruning task such as monitoring the process using either a smart phone or a tablet instead of a PC along with a more powerful wireless communication to cover a large working area. Moreover, a quadrotor helicopter in a coaxial configuration is also being considered to increase the payload capacity and thus allowing the operator to place an extra battery to increase the flying time, which is crucial to accomplish the pruning task.
